# Correlation between Positive Orientation and Control of Anger, Anxiety and Depression in Nursing Students in Poland, Spain and Slovakia during the COVID-19 Pandemic

**DOI:** 10.3390/ijerph19042482

**Published:** 2022-02-21

**Authors:** Ewa Kupcewicz, Marzena Mikla, Helena Kadučáková, Elżbieta Grochans, Maria Dolores Roldán Valcarcel, Anna Maria Cybulska

**Affiliations:** 1 Department of Nursing, Collegium Medicum University of Warmia and Mazury, 10-719 Olsztyn, Poland; ekupcewicz@wp.pl; 2 Department of Nursing, University of Murcia, Campus de Espinardo, 30100 Murcia, Spain; marzena.mikla@um.es (M.M.); mdrv1@um.es (M.D.R.V.); 3 Department of Nursing, Faculty of Health, Catholic University in Ruzomberok, 034-01 Ruzomberok, Slovakia; helena.kaducakova@ku.sk; 4 Department of Nursing, Pomeranian Medical University in Szczecin, 71-210 Szczecin, Poland; elzbieta.grochans@pum.edu.pl

**Keywords:** positive orientation, anxiety, depression, anger, nursing student

## Abstract

(1) This study analysed the relationship between positive orientation and subjective control of anger, anxiety and depression in nursing students in Poland, Spain and Slovakia during the pandemic. (2) The survey was carried out by the diagnostic survey method in a group of 756 nursing students in Poland, Spain and Slovakia. The empirical data were gathered using an original survey questionnaire, the Positive Orientation Scale and the Courtauld Emotional Control Scale. (3) It was shown that the positive orientation level in Polish students was significantly lower than in students in Spain (*p* < 0.0001) and Slovakia (*p* < 0.0001). Low results for positive orientation were obtained in nearly half (47.18%) of the respondents in the Polish sample, whereas it was 34.18% and 31.18% in the Spanish and Slovak samples, respectively (*p* < 0.0001). A positive orientation was the most important predictor of emotional control among the nursing students at all the sites (*p* < 0.0001). (4) Positive orientation has been shown to have a significant impact on emotional control in nursing students during the pandemic. Therefore, it is important to carefully monitor students’ mental health during the pandemic to determine the demand for psychological and emotional support.

## 1. Introduction

The COVID-19 pandemic outbreak in the first quarter of 2020 changed the world considerably. The previously unknown disease caused by the SARS-CoV-2 virus has contributed to people’s confrontation with potent stressors, which reduces mental performance and resistance with unprecedented consequences that are hard to estimate [1]. Anxiety and depression have been found to be the most frequent mental health issues [2,3,4].

Healthcare professionals and medical students are particularly susceptible to stress during this period [5]. Studying can be a challenge to many students as shown by research confirming a high incidence of anxiety and depression among students [6,7,8]. This applies mainly to medical students, who experience considerable stress caused by the extensive syllabuses, duration of studies and significant interference of studies with other areas of their lives [9,10,11,12]. Nursing students [13] are exposed to various stressors during their studies, which are directly linked to the clinical environment of studying, i.e., patient care, including the caring activities and medical procedures, contact with patients’ families and cooperation with the members of an interdisciplinary team [14]. Clinical internships or other activities are a key element of medical education, and they may have been restricted due to the pandemic.

A literature review found that there are many factors causing anxiety and depression in students. Age, sex and education level have a considerable impact on mental disorders [6,7,15,16].

Depression among nursing students was the object of numerous studies before the COVID-19 pandemic. One example is a study conducted by Cheung et al. of a group of 661 nursing students in Hong Kong. Depression was diagnosed in 35.8% of the study participants. It was also demonstrated that the study year, lack of physical exercise and a family crisis correlated significantly to depression. An unbalanced diet used by nursing students correlated significantly to anxiety [17]. It was found in another study conducted by Chernomas & Shapiro that 27% of nursing students in Canada suffered from mild to very severe depression [18]. Mechili et al. reported the findings of the first study conducted to evaluate the depression level in nursing and midwifery students (*n* = 863) and their family members (*n* = 249) when on quarantine during the COVID-19 pandemic in Albania and showed 25.2% (*n* = 217) of the students and 25.6% (*n* = 64) of their family members suffered from moderate to severe symptoms of depression [19]. The depression level in nursing students in Greece, Spain and Albania during the COVID-19 pandemic and identification of its possible determinants was also evaluated by Patelarou et al. The researchers found one-third of the group of 787 nursing students experienced mild depression, with a higher depression level being noted among students in Spain (59.1%), followed by those in Albania (34.5%) and Greece (21.8%). The students in Spain were found to experience depression more frequently than those in Greece or in Albania (*p* < 0.001) [20].

Despite a continuous expansion of the COVID-19 pandemic, nursing students have continued their study and training at university campuses and in a clinical environment. Masha’al et al. demonstrated that 70.6% of the nursing students in Jordan participating in the study experienced mild to severe anxiety after they returned to studies at their campus. The researchers confirmed a positive correlation between the anxiety level in the students and their dysfunctional coping strategies, which included denial, behavioural withdrawal, remorse and self-blaming [21]. Furthermore, the capability for behavioural adaptation and coping with stress, community support and optimism have a positive effect on students’ mental health [22,23,24,25,26].

Gonçalves et al. evaluated the correlation between religious and spiritual beliefs, anxiety and depression in medical and nursing students in Brazil. They found religiosity to be associated with a lower anxiety level in students, but not with depression [27]. Devi et al. demonstrated that immunity was a key mediator of the stress correlation to clinical practice and depression and anxiety in nursing students in Indonesia [28]. Nursing students often experience anger in response to stress, and they suppress their anger instead of controlling it actively [29]. Sharma et al. found that adopting adaptive behaviour helped nursing students to overcome anger [30].

Self-esteem, which determines how one perceives one’s worth, is another important factor. High self-esteem has a positive impact on one’s attitude to oneself, to others and to future goals and enhances positive emotions towards oneself, thereby reducing the level of stress and the risk of depression [31]. Higher self-esteem is associated with stronger expression of negative emotions, and lower self-esteem is associated with their suppression [32].

Caprara argues that positive orientation is largely responsible for the human adaptive function of a person. It denotes a natural inclination towards a favourable opinion about oneself, high satisfaction with life and being confident about the chances of attaining one’s goals, with consequent commitment in pursuing one’s goals and being satisfied with the quality of one’s life [33,34,35]. Empirically, a positive orientation is a hidden independent variable explaining the co-variation of self-esteem, satisfaction with life and optimism [33,34,35]. It has been confirmed in Canadian, German and Japanese studies that a positive orientation can be regarded as a “good functioning syndrome”, which is positively correlated to an assessment of one’s health status [35]. It has its roots in the biological system, which gives an emotional shade to human experiences [33,36]. Caprara et al. demonstrated that individuals with higher positivity rarely experienced depression symptoms [37]. During the process of their education, medical students prepare to fulfil their duties towards other people in health and sickness. For this reason, their mental well-being is important not only to themselves but also to the quality of healthcare that they are going to provide [38]. A literature review showed that the issue of positive orientation is rarely brought up in relation to coping with emotions and the students’ academic life. Therefore, based on theoretical assumptions and empirical data, it was decided that the presented variables would demonstrate correlations.

The aim of this study was to determine the correlation between a positive orientation and subjective control of anger, anxiety and depression in nursing students in Poland, Spain and Slovakia during the COVID-19 pandemic.

The aim will be achieved by answering the following research questions:

- What level of positive orientation do nursing students declare, and to what extent do they control anger, depression and anxiety, taking into account the inter-group differences?
- To what extent is the declared level of positive orientation connected with the subjective control of anger, depression and anxiety in nursing students and what are the inter-group differences?
- What is the role of positive orientation, and which of the socio-demographic factors under study have greater predictive importance in emotional control in nursing students, taking into account the inter-group differences?

## 2. Materials and Methods

### 2.1. Settings and Design

The study was conducted in a group of 756 nursing students in first degree (bachelor degree) studies between 20 March and 15 May 2021. After consent was obtained from the dean, a survey was carried out among students of the University of Warmia and Mazury in Olsztyn, Pomeranian Medical University in Szczecin (Poland), as well as in Murcia University (Spain) and in the Catholic University in Ružomberok (Slovakia). Student age under 30 years was the enrolment criterion, and students who refused to grant their informed and voluntary consent for participation in the study were excluded. Because of the pandemic, the paper-and-pencil method and the online method was used. The survey in Poland and in Spain was carried out at the university, during classes, after agreeing on the survey date and time with the respective teachers. The questionnaire sets were distributed by one researcher at each of the universities while maintaining the sanitary regime due to the COVID-19 pandemic. Due to the sanitary restrictions and the fact that classes were conducted online, questionnaire sets were sent to the Slovak university students by e-mail and then, completed, they were returned by the same route within two days. The students were provided with exhaustive information on the study; they could ask questions and receive answers. Participation in the study was voluntary, and the respondents could withdraw at any time without giving a reason. Data confidentiality and students’ right to privacy was ensured. It took approx. ten minutes, on average, to complete the questionnaire.

A total of 850 survey forms were distributed. After the material was collected and those completed incorrectly were eliminated, 756 (88.94%) were taken for the statistical analysis. The empirical data were collected in the database and encoded with Excel, and the results were analysed collectively. All safety procedures of obtaining, storing and processing the data were followed with due diligence.

The research project was given a favourable opinion (No. 3/2021) by the Senate Scientific Research Ethics Committee at the Olsztyn University in Olsztyn and was carried out in accordance with the Declaration of Helsinki and the procedures and instructions in force in the universities. This study is part of an international research project executed as part of one of the researcher’s (E.K.) scientific internship programmes.

### 2.2. Participants

The study included 756 nursing students from three European countries: Poland—390 (51.59%), Spain—196 (25.92%), Slovakia—170 (22.49%). The mean age of the participants was 21.20 years (SD = 1.97). There were 29.50% (*n* = 223) first-year students, 38.89% (*n* = 294) second-year students and 31.61% (*n* = 239) third-year students. According to ¾ of the study participants, their physical activity was greatly restricted as a consequence of the SARS-CoV-2 virus spread. Almost all the participants described their health status as very good or good. The students usually had three (*n* = 266; 35.19%) or four (*n* = 293; 38.76%) meals daily, but they had only some of them at fixed times. A great majority of the participants declared that their social contacts during the COVID-19 pandemic were restricted to a great or average extent. Due to the online classes, the students spent a lot of time in front of the computer. One-third of the students in Spain (31.12%) and one-fifth of those in Poland (20.77%) declared that it was ≥10 h (Table 1).

### 2.3. Research Instruments

The diagnostic survey method was applied using the questionnaire technique, and standardised research tools were used to collect the empirical data:

- Positive Orientation Scale by G.V. Caprara et al., adapted by Łaguna et al. [35];
- Emotional Control Scale—CECS by M. Watson, S. Greer, adapted by Z. Juczyński [32].

An original survey questionnaire was used to determine the socio-demographic factors i.e., place of residence, gender, age, study year, as well as a subjective evaluation of the health status and selected elements of the lifestyle during the COVID-19 pandemic (i.e., physical activity, meals, time spent working on a computer).

#### 2.3.1. Positive Orientation Scale (P-Scale)

The P-Scale measures positive orientation as the main tendency to notice positive aspects of oneself, one’s own experience and life. The scale includes eight diagnostic statements, and study participants are asked to indicate the extent to which they agree with each of them.

The answers are given on a 5-degree Likert scale, from 1 to 5 (1—I definitely disagree, 5—I definitely agree, with one statement (4) being reversed). The total score obtained in the test gives a positive orientation coefficient for the participants. The higher the score, the higher the positive orientation level, the raw scores range from 8 to 40 points. After being converted to standardised units, the raw scores are interpreted according to the properties characterising the sten scale. The scores between 7 and 10 sten were regarded as high, 5 and 6—as average, and those from 1 to 4 sten as low. The P-scale has a single-factorial structure, it has sufficient internal consistency (Cronbach alpha = 0.77–0.84), constancy (rtt = 0.84) and confirmed convergent validity [35].

#### 2.3.2. CECS—Courtauld Emotional Control Scale

The CECS scale is used to measure the subjective control of anger, anxiety and depression in difficult situations. It consists of three subscales, each of which includes seven statements on the methods of showing anger, anxiety and depression.

A participant noted how often a way of showing their emotions occurs, on a four-point scale, from “hardly ever” (1 point) to “nearly always” (4 points). The results were calculated for each subscale. Before the calculations were made, which involved the summing up of points in each of the three subscales, the total points given for the statement expressing forms of revealing (rather than suppressing) emotions were changed. The score was reversed to 4-3-2-1 for the following statements: on the scale for anger—2, 4; on the scale for depression—1, 5; on the scale for anxiety—1, 4, 5. The score for each of the scales ranged from 7 to 28 points. The sum of all the three subscales gave a total emotion control index, which denoted an individual’s subjective conviction about their ability to control their responses to the specific negative emotions. The total emotion control index ranged from 21 to 84 points. A higher score represented a higher degree of the negative emotion suppression. The scale reliability was assessed by estimating its internal consistency, and the following Cronbach alpha coefficients were calculated: 0.80 for anger control; 0.77 for depression control; 0.78 for anxiety control and 0.87 for the total emotion control index. The internal consistency coefficients for the original scale version were higher: 0.86 for anger; 0.88 for depression and 0.88 for anxiety [32].

### 2.4. Statistical Analysis

A statistical analysis of the data was performed with the Polish version of STATISTICA 13 (TIBCO, Palo Alto, CA, USA). The respondents were characterised by the number and %, and the group equipotency was verified with the chi-square test (χ^2^). The statistical data obtained from the study were described with the mean (M), median (Me), minimum–maximum (Min.–Max.), standard deviation (SD) and 95% confidence interval (CI).

The diversity of results for positive orientation and control of emotions (anger, depression, anxiety) was evaluated by means of the analysis of variance (ANOVA) (F) for a comparison of multiple samples in independent groups, and it was detailed with a post-hoc test (LSD). An analysis of the correlation strength between positive orientation and emotional control was performed with Pearson’s correlation (r). The emotional control predictors were sought with a multiple regression analysis in order to build a random variable estimation model from the independent variables. The interpretation of the correlation strength between the analysed variables was based on Guilford’s classification, taking, in sequence: |r| = 0—no correlation, 0.0 < |r|≤ 0.1—slight correlation, 0.1 < |r| ≤ 0.3—weak correlation, 0.3 < |r| ≤ 0.5—average correlation, 0.5 < |r| ≤ 0.7—high correlation, 0.7 < |r|≤ 0.9—very high correlation, 0.9 < |r| < 1.0—nearly full correlation, |r|=1—full correlation [39]. The test probability at the level of significance of *p* < 0.05 was regarded as significant. The study meets the criteria of a cross-sectional study [40].

## 3. Results

### 3.1. Diversity of Results for Positive Orientation and Emotional Control in Students in the Polish, Spanish and Slovak Studies

The diversity of results for positive orientation and emotional control in students in the Polish, Spanish and Slovak studies was evaluated. A statistical analysis with the analysis of variance ANOVA test showed a statistically significant diversity of the mean for positive orientation in the subgroups, which manifested itself as a general tendency for perceiving life experiences with a positive attitude (F = 8.30; *p* < 0.0002). The analysed mean scores for positive orientation in the Polish sample were 27.53 (SD = 6.01) points on a scale from 8 to 40 points, and 29.20 (SD = 5.18) and 29.22 (SD = 5.49) points in the Spanish and Slovak samples, respectively (Table 2).

**Figure 1:**
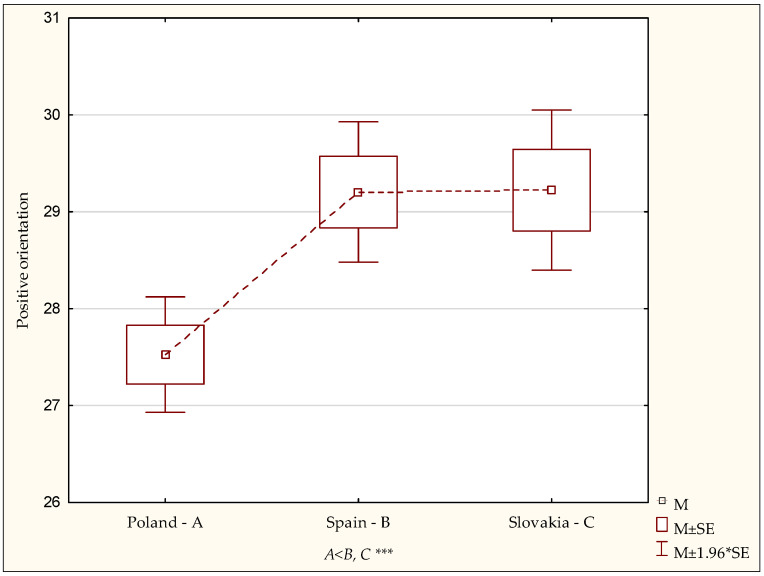
Positive orientation—diversity of results in the Polish, Spanish and Slovak studies. Statistically significant: *** *p* < 0.001.

In the next step of the analysis of variance, a post-hoc test (LSD) was used, whose results showed that the positive orientation level in students in Poland was significantly lower than in students in Spain (*p* < 0.0001) and Slovakia (*p* < 0.0001) (Figure 1).

The diversity of scores for positive orientation on the sten scale in students in the Polish, Spanish and Slovak studies was evaluated. Significant diversity was observed for low, average and high scores in the subgroups under study (χ^2^ = 17.01; *p* < 0.0001). The low scores for the positive orientation were obtained in nearly half (47.18%) of the respondents in the Polish sample, whereas it was 34.18% and 31.18% in the Spanish and Slovak studies, respectively. The highest percentage of high scores was noted in the Slovak sample (36.47%), and the lowest was in the Polish sample (26.41%) (Table 3).

Table 2 shows data on emotional control in general and is broken down into subscales (control of anger, anxiety and depression). The ANOVA did not reveal any significant differences regarding emotional control in the general dimension between the nursing students subgroups under study. The mean results for emotional control, which denote the nursing students’ subjective conviction about their ability to control their responses to the specific negative emotions, were 50.94 (SD = 12.65) points on a scale from 21 to 84 points. They were slightly higher, but not statistically significant, in the Spanish (M = 51.40; SD = 9.32) and Slovak samples (M = 52.69; SD = 12.06).

**Figure 2:**
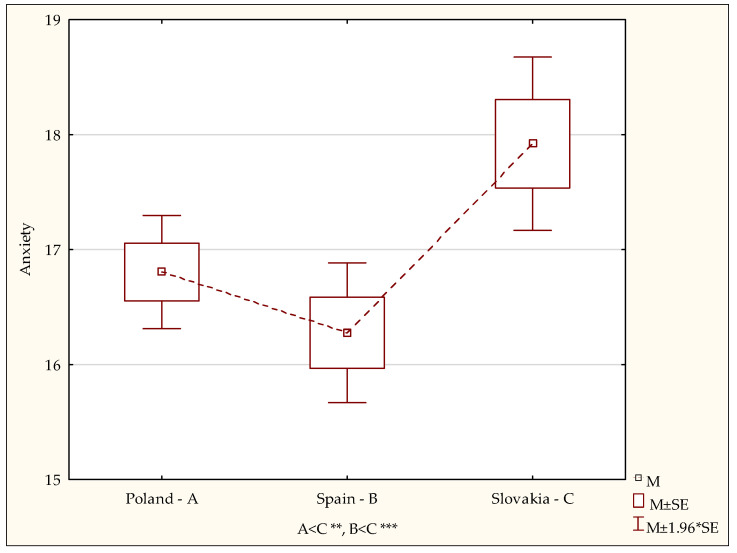
Diversity of scores in the anxiety control subscale in the Polish, Spanish and Slovak studies. Statistically significant: ** *p* < 0.01; *** *p* < 0.001.

A diversity of indices for control of anger and depression in the samples under study, considering the country of residence, was not confirmed. This indicates that nursing students exhibit a similar general tendency for suppressing negative emotions and for subjective control of anger and depression regardless of their place of residence. A statistically significant diversity between groups was observed only for the anxiety control indices (F = 5.51; *p* < 0.004). It was shown in detailed analyses by the post-hoc (LSD) test that the anxiety control index for the Polish students was significantly lower than in the Slovak study (*p* < 0.0001). Similarly, the index for the Spanish students was also significantly lower than in the students in Slovakia (*p* < 0.0001) (Figure 2). This means that students in Poland and Spain demonstrate a lower tendency for emotional suppression for anxiety, whereas students in Slovakia displayed increased anxiety control during the COVID-19 pandemic.

### 3.2. The Strength of the Correlation between Positive Orientation and Anger, Depression and Anxiety Control in Students in the Polish, Spanish and Slovak Studies

The study assessed the strength of correlation between positive orientation and anger, depression and anxiety control in students in the Polish, Spanish and Slovak studies. The Pearson correlation (r) analysis revealed a statistically significant correlation between positive orientation and a general coefficient of emotional control, as well as anger, depression and anxiety control in students in the Polish, Spanish and Slovak studies.

Positive orientation is negatively correlated, with various strengths, to all the spheres of emotion control and its overall coefficient, which means that higher levels of positive orientation are associated with stronger expression of negative emotions and their lower levels are associated with emotion suppression.

**Figure 3:**
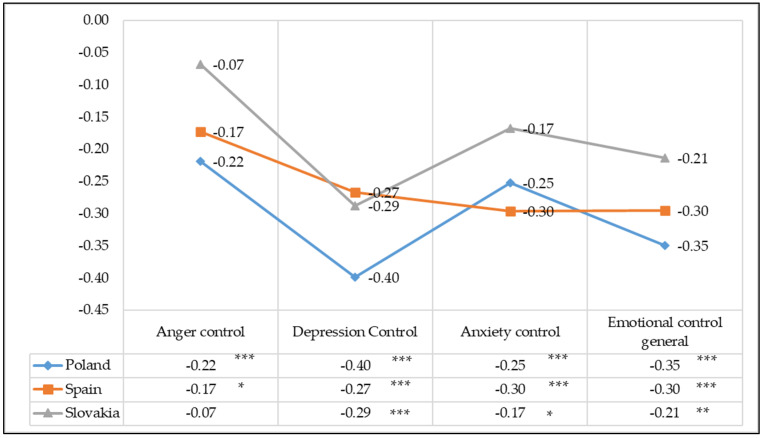
The nature and strength of the correlation between positive orientation and anger, depression and anxiety control in students—Pearson correlation coefficient (r). Statistically significant: * *p* < 0.05; ** *p* < 0.01; *** *p* < 0.001.

According to the findings of this study, positive orientation is negatively correlated at an average level with the general index of emotional control in the Polish (r = −0.35; *p* < 0.0001) and Spanish (r = −0.30; *p* < 0.0001) samples, whereas the correlation strength is low in the Slovak sample (r = −0.21; *p* < 0.0001). An analysis of the nature and strength of correlation between positive orientation and emotional control in its individual dimension showed the greatest correlation strength for depression control, which was negative on an average level (r = −0.40; *p* < 0.0001) in the Polish sample and for anxiety control in the Spanish sample (r = −0.30; *p* < 0.0001). The correlation between positive orientation and anger control is significantly negative in all samples, and their strength, according to Guilford’s classification, is weak or slight. (Figure 3).

### 3.3. General Predictors of Emotional Control of Anger, Depression and Anxiety

A multiple regression analysis was applied to determine the share of positive orientation and the socio-demographic factors as well as selected lifestyle elements in determining the diversity of general emotional control, anger, depression and anxiety in the nursing students under study.

Two groups of factors were identified in the process of the regression model development. The group of dependent variables (y) included the general emotion control index and the anger, depression and anxiety control indices. The independent variables (x) included initially: positive orientation, age, gender, study year, place and form of residence, time spent working on a computer, regularity of meals, reduction of social contacts during the pandemic, a subjective health status assessment and physical exercise restriction during the pandemic. However, two factors: gender and physical exercise restriction during the pandemic, were excluded from the pool of the independent variables (y) under analysis as a result of the statistical analyses.

#### 3.3.1. Multiple Regression Results in the Polish Study

The multiple regression analysis results clearly show that positive orientation is the main predictor of emotional control, both in general and with respect to anger, depression and anxiety control in the Polish sample. Its predictive strength was most marked in depression control, as a positive orientation explained 16% of the results’ variation (βeta = −0.35; R^2^ = 0.17). The second factor concerning the time spent working on a computer in the depression control explained 1% of the results’ variation, and it had only slight significance. According to the regression analysis results, anger control was explained by only one factor—positive orientation at 5% of variance (βeta = −0.23; R^2^ = 0.05), whereas anxiety control was explained by three factors at 8% of the results’ variation, including positive orientation 6% (βeta = −0.26; R^2^ = 0.08), the other two factors (age and regularity of meals) did not play a significant role. In the case of general emotional control, positive orientation explained 12% of the results’ variation (βeta = −0.34; R^2^ = 0.12) (Table 4).

#### 3.3.2. Multiple Regression Results in the Spanish Study

An analysis of data in Table 5 indicates that two factors were predictors of general emotional control among nursing students in Spain, which in total explained 11% of the variation of the results, including positive orientation, which explained 9% (βeta = −0.30; R^2^ = 0.11), and the respondents’ age—2%. The factors—positive orientation and reduced social contacts during the COVID-19 pandemic—were determinants of anger control, which in total explained 6% of the variation (βeta = −0.20; R^2^ = 0.06), 3% each. In the case of depression control, positive orientation was in first place as a predictor, which explained 7% of the results’ variation (βeta = −0,24; R^2^ = 0,09), followed by the students’ place and form of residence during the COVID-19, explaining 2% of the results’ variation. Moreover, two factors were identified—positive orientation and the respondents’ age for the anxiety control, which together explained 10% of the variation. Positive orientation played the most important role in the prediction of anxiety control in the group of Spanish students at 9% (βeta = −0.31; R^2^ = 0.10).

#### 3.3.3. Multiple Regression Results in the Slovak Study

The regression analysis results show that the independent factor model in the Slovak sample revealed a subjective health status assessment by the students during the COVID-19 pandemic and positive orientation as predictors of emotional control. In general emotional control, both of the identified factors explained 8% of the results’ variation, and the health status assessment explained 5% of the variation (βeta = −0.21; R^2^ = 0.05). Only the subjective health status assessment by Slovak students turned out to be a predictor, explaining 4% of the results’ variation (βeta = −0.25; R^2^ = 0.04).

In turn, positive orientation was the main independent variable in depression control, explaining 8% of the results’ variation (βeta = −0.28; R² = 0.04). Subjective health status assessment during the COVID-19 pandemic played a minor role, explaining only 2% of the variation.

Positive orientation explained 3% of the results variation in anxiety control (βeta = −0.20; R^2^ = 0.03) (Table 6).

In conclusion, positive orientation was the most important predictor of emotional control among nursing students. All regression results (Table 4, Table 5 and Table 6) were statistically significant, and the regression coefficients were negative, indicating a negative correlation, which means that a higher positive orientation level is associated with stronger expression of negative emotions, while a lower level is associated with their suppression.

## 4. Discussion

The findings of many studies show that emotion suppression leads to their amplification or contributes to their prolonged maintenance as emotional tension [32]. Emotions vary with respect to the ways of their expression and experience. The overall emotion control indices as determined in this study among the students in Poland, Spain and Slovakia ranged from 50.94 to 52.69 points and were higher than in the normalisation study (48.59) for the group aged 20–30 years [32]. It is also noteworthy that a normalisation study revealed considerable differences in the emotion control indices in several clinical groups [32]. For example, the mean general emotion control index for patients on dialysis was 51.23, and for menopausal women it was 51.84, which was comparable to the findings of the current study, whereas it was much higher in diabetics (55.77) and males after cardiac infarction (53.03) [32]. Increased emotion control is a clear indication that these groups should be provided with special psychological care.

The study helped to determine the indices for the control of three negative emotions, i.e., anger, depression and anxiety. The authors also sought a correlation between positive orientation and the emotions under examination in nursing students. It was found in this study that the anger control indices were higher in all groups than in the normalisation study [32]. This means that the nursing students participating in the study tend to suppress anger. This emotion is often associated with the repeated impact of stressors. A person exposed to stressors and unable to respond to them vents his/her anger [41,42]. Threats associated with the COVID-19 pandemic and the restrictions imposed because of the spread of infections have become stressors not only to nursing students but to the whole global population. Myers points out that controlled expression of anger can be more adaptive than an outbreak of anger [42]. The literature review shows that nursing students often experience anger in response to stress and suppress their anger rather than control it actively [43,44]. Jun et al. demonstrated that it is necessary for nursing students to participate in an anger control programme to acquire the ability to cope with it [43]. Social support can also help nursing students to cope effectively with anger, which they experience in consequence of life stress [44]. Many researchers have pointed out that anger control programmes based on cognitive behavioural therapy effectively improve adaptive anger expression [45,46,47,48].

The depression control indices determined in this study were higher than in the normalisation study [32]. The results show that the severity and experience of depressive symptoms among nursing students during the COVID-19 pandemic is worrying and probably requires systemic action.

On the other hand, the anxiety control indices in the Polish (16.81) and the Spanish groups (16.28) were lower than in the normalisation study (17.12) [32]. The higher level of anxiety suppression among the students in Slovakia, as observed in this study, may result from an inability to verbalise feelings.

Students are a population particularly susceptible to mental health issues. Several studies have confirmed that students of medicine, nursing, pharmacy, midwifery, dentistry and physiotherapy exhibit an increased incidence of the symptoms of depression [49,50,51,52] and anxiety [53,54,55,56] compared to students of non-medical majors.

Zhang et al. demonstrated that [57] the incidence of negative emotions, especially anxiety, increased considerably compared to earlier studies among Chinese students during the initial phase of the COVID-19 pandemic [58,59]. The growing trend in negative emotions may suggest that the negative impact of COVID-19 on public mental health will increase as the disease spreads around the world. Nakhostin-Ansari A et al. [60] also noted a definitely higher incidence of anxiety among Iranian medical students (38%) compared to Chinese students [59]. Fu W. et al. [61] observed that approximately two-fifths of Chinese college students experienced anxiety symptoms during the COVID-19 pandemic.

Ochnik et al. conducted an international study aimed at exploring differences in mental health status among university students in nine countries (Poland, Ukraine, Turkey, Columbia, Czechia, Germany, Israel, Russia and Slovenia) during the first wave of the COVID-19 pandemic and revealed differences between them in terms of depression and anxiety. The students in Turkey were the most exposed to a risk of depression and anxiety. The lowest depression indices were noted among the students in Czechia, and the lowest anxiety indices were among those in Germany. Significantly higher scores regarding the analysed variables were noted among students in Poland than in Czechia, Slovenia, Ukraine and Germany, and also among those in Russia—regarding the anxiety scores [62]. Kalkan Uğurlu et al. also demonstrated a negative impact of the COVID-19 pandemic on mental health and nutritional behaviours in nursing students in Turkey [63]. An interesting study was conducted by Gotlib et al. in a group of 790 nursing students in Poland, in which the researchers sought a correlation between the level of anxiety among the students and their knowledge of COVID-19 vaccines and between the level of anxiety, the knowledge of the vaccinations and the will to vaccinate against COVID-19. It turned out that the anxiety level among the nursing students was low, with no direct correlation between the knowledge of the vaccines and the anxiety level [64].

The general emotion control index and the scores in the anger, depression and anxiety subscales as determined in this study correlate negatively to the positive orientation, which confirms that a higher sense of positive orientation in nursing students is associated with stronger expression of negative emotions and a lower sense is associated with their suppression.

A study conducted by Shi et al. [65] also showed that there were three psychological variables: resistance, hope and optimism, which were correlated negatively with depression symptoms among Chinese medical students. It was found in the study by Tian et al. [66] that the score on the P-scale was a significant predictor for subjective well-being and depression. A study conducted by Günaydın HD. [67] analysed the impact of the ability to solve social problems on academic motivation by means of the fear of COVID-19. It was found that a positive orientation was regarded as a significant psychological source of strength to protect oneself against COVID-19-related mental issues. It helps to reduce the stress related to everyday academic hardships and boosts one’s motivation to cope effectively with more difficult projects.

A study conducted by Bodys-Cupak et al. among Polish nursing students [68] showed a high and average level of optimism in the majority of the study participants. Those with a higher level of dispositional optimism coped with stress and negative emotions significantly more frequently. These findings were consistent with those of a study by Joseph N. et al. [69]. Moreover, a study by Kupcewicz et al. [70] conducted among Polish nurses confirmed that a decreased level of positive orientation proved to be the main determinant of professional burnout among them.

A literature review showed that positivity could be significantly linked to life satisfaction, self-esteem, optimism and symptoms of depression [37], happiness [71] and resistance [72]. Evidence also suggests that people with psychological resources, possibilities and strengths, such as positive emotions (e.g., happiness), positive individual features (e.g., positivity) and social environmental factors (e.g., community support), can maintain positive mental health [73].

In conclusion to these considerations, two important studies should be noted which show how difficult and responsible a role is played by nursing students during the COVID-19 pandemic. The global health and social crisis overwhelmed some healthcare systems in the early stages of the COVID-19 pandemic, which was caused mainly by staff shortages. In consequence, healthcare facilities started to hire nursing students. Casafont et al. examined how final-year nursing students in Spain perceived experiences related to their employment to help the National Healthcare System in response to the COVID-19 pandemic. The researchers noted that nursing students faced many adversities during their work with patients caused by the work environment. They also mentioned adaptive and emotional issues [74].

The research conducted by Rodríguez-Almagro et al. also demonstrated that nursing students were tired and mentally exhausted by working for several days in a row when employed as healthcare workers. Nevertheless, they managed to overcome all the difficulties and to demonstrate their professional commitment and resistance [75]. According to the authors of both studies, emotional support in crisis situations is the key to overcoming stressful and emotional situations by inexperienced healthcare staff [74,75].

Referring to the current situation of nursing students during the COVID-19 pandemic, it should be noted that similar psychological support should be provided to graduate nurses who have recently started work and have little professional experience and are more exposed to stressors in the work environment than those with longer experience. This is supported by the results of a study conducted in Spain by Del Pozo-Herce et al. [76].

In conclusion, it is difficult to evaluate the overall impact of the university environment on students’ mental health. However, given the growing incidence of emotional disorders among students during the COVID-19 pandemic, one can expect this situation to have a significant impact on this population. The pandemic has shown that many students, especially medical students, experience considerable psychological symptoms associated with anxiety, stress and depression [60]. It is therefore important to develop new guidelines for consulting and online psychological interventions to provide support to future medical personnel and to promote a positive orientation among students.

## 5. Study Limitations

Several limitations of this study should be taken into account. Firstly, this study is a cross-sectional study in which positive orientation is assessed as a latent variable. Secondly, all data were obtained using self-reported questionnaires, which could have introduced response bias. Participants may have underestimated or overestimated their ability to control anger, anxiety and depression. Finally, restrictions connected with the COVID-19 pandemic made it difficult to reach students and recruit a larger study group.

## 6. Conclusions

- This study found that a positive orientation was the most important predictor of emotional (anger, depression, anxiety) control among nursing students. The higher the positive orientation level among students, the less prone they are to suppress anger, anxiety and depression.
- The study also showed that the positive orientation index in nursing students in Poland was lower than in such students in Spain and Slovakia.
- According to the findings of this study, positive orientation is negatively correlated at an average level with a general index of emotional control in the Polish and Spanish samples. A strong correlation was observed between positive orientation and depression control among nursing students in Poland and between positivity and anxiety control in the Spanish sample. There was a strong correlation between positive orientation and anger control among all of the students (Poland, Spain, Slovakia).
- Positive orientation was shown to have a significant impact on emotional control in nursing students during the COVID-19 pandemic. Therefore, it is important to carefully monitor students’ mental health during the pandemic to determine the demand for psychological and emotional support.

## Figures and Tables

**Table 1:** Characteristics of the studied group.

Variables		Total N = 756	Country of Residence			Chi-Square Test χ^2^	*p*
			Poland—A *n* = 390 (51.59%)	Spain—B *n* = 196 (25.92%)	Slovakia—C *n* = 170 (22.49%)		
		*n* (%)	*n* (%)	*n* (%)	*n* (%)		
Gender	female	682 (90.21)	357 (91.54)	161 (82.14)	164 (96.47)	22.48	0.0001 ***
	male	74 (9.79)	33 (8.46)	35 (17.86)	6 (3.53)		
Study year	first	223 (29.50)	140 (35.90)	28 (14.29)	55 (32.35)	50.59	
	second	294 (38.89)	160 (41.03)	73 (37.24)	61 (35.88)		
	third	239 (31.61)	90 (23.08)	95 (48.47)	64 (37.65)		
Age (years)	≤20	296 (39.15)	128 (51.28)	63 (32.14)	71 (41.76)	26.62	0.0001 ***
	21–22	334 (44.18)	200 (44.18)	29 (14.80)	36 (20.59)		
	≥23	126 (16.67)	62 (15.90)	126 (16.67)	126 (16.67)		
Place and form of residence	with family/ someone close	630 (83.33)	297 (76.15)	192 (97.96)	141 (82.94)	58.42	0.0001 ***
	on their own	126 (16.67)	93 (23.85)	4 (2.04)	29 (17.06)		
Time of work on a computer (hours)	≤5	327 (43.25)	174 (44.62)	51 (26.02)	102 (60.00)	56.33	0.0001 ***
	6–9	274 (36.24)	135 (34.62)	84 (42.86)	55 (32.35)		
	≥10	155 (20.50)	81 (20.77)	61 (31.12)	13 (7.65)		

Statistically significant: *** *p* < 0.0001. Explanation: N—number of group members; *n*—number of subgroup members.

**Table 2:** Diversity of results for positive orientation and emotional control in students in the Polish, Spanish and Slovak studies.

Country of Residence	Variables	P-Scale	CECS—General	CECS—Subscales		
				Anger Control	Depression Control	Anxiety Control
Poland—A *n* = 390 (51.59%)	M, SD	27.53, 6.01	50.94, 12.65	16.31, 5.31	17.83, 5.05	16.81, 4.96
	Me	28	50	16	18	17
	Min.–Max.	9–39	21–84	7–28	7–28	7–28
	95% Cl	26.93–28.12	49.68–52.20	15.78–16.83	17.33–18.34	16.31–17.30
Spain—B *n* = 196 (25.92%)	M, SD	29.20, 5.18	51.40, 9.32,51	17.18, 3.36	17.94, 3.33	16.28, 4.34
	Me	30	65	17	18	17
	Min.–Max.	12–40	29–75	10–25	11–25	7–28
	95% Cl	28.47–29.93	50.09–52.71	16.71–17.66	17.47–18.41	15.67–16.89
Slovakia—C *n* = 170 (22.49%)	M, SD	29.22, 5.49	52.69, 12.06	16.89, 4.87	17.88, 4.87	17.93, 5.01
	Me	30	53	17	18	18
	Min.–Max.	12–40	21–81	7–28	7–28	7–28
	95% Cl	28.39–30.06	50.87–52.29	16.15–17.63	17.14–18.62	17.16–18.68
ANOVA (F)		8.30	1.31	2.44	0.03	5.51
*p*-value		0.0002 A < B, C ***	0.26	0.08	0.96	0.004 A < C **, B < C ***

Statistically significant: ** *p* < 0.01; *** *p* < 0.001. Explanation: P-scale—Positive Orientation Scale, CECS—Emotional Control Scale, M—mean, SD—standard deviation, Me—median, Min.—minimum, Max.—maximum, 95% confidence interval (CI)

**Table 3:** Percentage of scores for positive orientation on the sten scale in the Polish, Spanish and Slovak studies.

Variables	Scores on the Sten Scale (1–10 sten)	Country of Residence			Chi-Square Test χ^2^	*p*-Value
		Poland—A *n* = 390 (51.59%)	Spain—B *n* = 196 (25.92%)	Slovakia—C *n* = 170 (22.49%)		
		*n* (%)	*n* (%)	*n* (%)		
Positive orientation	Low (1–4)	184 (47.18)	67 (34.18)	53 (31.18)	17.01	0.001 ***
	Average (5–6)	103 (26.41)	64 (32.65)	55 (32.35)		
	High (7–10)	103 (26.41)	65 (33.16)	62 (36.47)		

Statistically significant: *** *p* < 0.001. *n*—number of participants in a subgroup.

**Table 4:** Multiple regression results—predictors of emotional control—general, concerning anger, depression and anxiety in the Polish study.

Factors under Analysis		Summary			
		R^2^	βeta	β	*p*-Value
Emotional control— general	Constant value			62.65	0.0001 ***
	Positive orientation	0.12	−0.34	−0.72	0.0001 ***
	R = 0.37; R^2^ = 0.14; corrected R^2^ = 0.12				
Anger control	Constant value			17.76	0.0004 ***
	Positive orientation	0.05	−0.23	−0.21	0.0004 ***
	R = 0.23; R^2^ = 0.06; corrected R^2^ = 0.05				
Depression control	Constant value			28.82	0.0001 ***
	Positive orientation	0.16	−0.35	−0.29	0.0001 ***
	Time of work on a computer	0.17	0.09	0.14	0.07
	R = 0.42; R^2^ = 0.17; corrected R^2^ = 0.17				
Anxiety control	Constant value			15.24	0.0003 ***
	Positive orientation	0.06	−0.26	−0.21	0.0001 ***
	Age	0.07	0.11	0.28	0.03 *
	Regular meals	0.08	0.10	0.79	0.04 *
	R = 0.30; R^2^ = 0.09; corrected R^2^ = 0.08				

Statistically significant: * *p* < 0.05; *** *p* < 0.001.

**Table 5:** Multiple regression results—predictors of emotional control—general, concerning anger, depression and anxiety in the Spanish study.

Factors under Analysis		Summary			
		R^2^	βeta	β	*p*-Value
Emotional control— general	Constant value			46.26	0.0001 ***
	Positive orientation	0.09	−0.30	−0.54	0.0001 ***
	Age	0.11	0.12	0.54	0.09
	R = 0.36; R^2^ = 0.12; corrected R^2^ = 0.11				
Anger control	Constant value			19.35	0.0001 ***
	Positive orientation	0.03	−0.20	−0.13	0.01 *
	Reduction of social contacts during pandemic	0.06	−0.16	−0.59	0.02 *
	R = 0.29; R^2^=0.08; corrected R^2^ = 0.06				
Depression control	Constant value			12.71	0.0004 ***
	Positive orientation	0.07	−0.24	−0.15	0.001 ***
	Place and form of residence	0.09	0.13	3.05	0.07
	R = 0.34; R^2^ = 0.12; corrected R^2^ = 0.09				
Anxiety control	Constant value			13.07	0.002 ***
	Positive orientation	0.09	−0.31	−0.26	0.0001 ***
	Age	0.10	0.15	0.33	0.03 *
	R = 0.35; R^2^ = 0.12; corrected R^2^ = 0.10				

Statistically significant: * *p* < 0.05; *** *p* < 0.001.

**Table 6:** Multiple regression results—predictors of emotional control—general, concerning anger, depression and anxiety in the Slovak study.

Factors under Analysis		Summary			
		R^2^	βeta	β	*p*-Value
Emotional control— general	Constant value			74.14	0.0001 ***
	Subjective health status assessment during the pandemic	0.05	−0.21	−5.13	0.01 **
	Positive orientation	0.07	−0.20	−0.44	0.01 **
	R = 0.32; R^2^ = 0.10; corrected R^2^ = 0.08				
Anger control	Constant value			20.77	0.0001 ***
	Subjective health status assessment during the pandemic	0.04	−0.25	−2.53	0.002 ***
	R = 0.29; R^2^ = 0.09; corrected R^2^ = 0.04				
Depression control	Constant value			31.42	0.0001 ***
	Positive orientation	0.08	−0.28	−0.24	0.001 ***
	Subjective health status assessment during the pandemic	0.10	−0.17	−1.70	0.04 *
	R = 0.37; R^2^ = 0.14; corrected R^2^ = 0.10				
Anxiety control	Constant value			25.30	0.0001 ***
	Positive orientation	0.03	−0.20	−0.18	0.02 *
	R = 0.23; R^2^ = 0.05; corrected R^2^ = 0.03				

Statistically significant: * *p* < 0.05; ** *p* < 0.01; *** *p* < 0.001.

## Data Availability

The data presented in this study are available on request from the first author.
